# High Performance Complementary Circuits Based on *p*-SnO and *n*-IGZO Thin-Film Transistors

**DOI:** 10.3390/ma10030319

**Published:** 2017-03-21

**Authors:** Jiawei Zhang, Jia Yang, Yunpeng Li, Joshua Wilson, Xiaochen Ma, Qian Xin, Aimin Song

**Affiliations:** 1 School of Electrical and Electronic Engineering, University of Manchester, Manchester M13 9PL, UK; jiawei.zhang@manchester.ac.uk (J.Z.); joshua.wilson@manchester.ac.uk (J.W.); xiaochenma0531@gmail.com (X.M.); 2 School of Physics, Shandong University, Jinan 250100, China; 18253166531@163.com (J.Y.); ypli2013@126.com (Y.L.)

**Keywords:** thin-film transistor, ring oscillators, IGZO, SnO

## Abstract

Oxide semiconductors are regarded as promising materials for large-area and/or flexible electronics. In this work, a ring oscillator based on *n*-type indium-gallium-zinc-oxide (IGZO) and *p-*type tin monoxide (SnO) is presented. The IGZO thin-film transistor (TFT) shows a linear mobility of 11.9 cm^2^/(V∙s) and a threshold voltage of 12.2 V. The SnO TFT exhibits a mobility of 0.51 cm^2^/(V∙s) and a threshold voltage of 20.1 V which is suitable for use with IGZO TFTs to form complementary circuits. At a supply voltage of 40 V, the complementary inverter shows a full output voltage swing and a gain of 24 with both TFTs having the same channel length/channel width ratio. The three-stage ring oscillator based on IGZO and SnO is able to operate at 2.63 kHz and the peak-to-peak oscillation amplitude reaches 36.1 V at a supply voltage of 40 V. The oxide-based complementary circuits, after further optimization of the operation voltage, may have wide applications in practical large-area flexible electronics.

## 1. Introduction

Oxide semiconductors have received much attention for a wide range of emerging applications such as flexible screens and wearable electronics [1,2]. Compared with conventional thin-film semiconductors such as amorphous silicon, they have a number of advantages including high electron mobilities, low fabrication temperatures, scalable deposition methods, highly uniform surfaces, and mechanical flexibility [3]. The desirability of oxide semiconductors is furthered by their large band gap which allows for high optical transmittance in the visible spectrum, a prerequisite for transparent electronics [2].

So far, vast progress has been made in *n*-type oxide semiconductors such as ZnO and amorphous InGaZnO_x_ (IGZO). For instance, IGZO has started to be commercialized to replace amorphous silicon for backplane drivers of flat-panel displays [4]. Schottky diodes [5,6] and thin-film transistors (TFTs) [7] based on IGZO have also demonstrated operating frequencies in the gigahertz regime. In contrast, there are relatively few studies on *p*-type oxide semiconductors. *P*-channel TFTs are necessary in order to fabricate high-performance CMOS logic gates for practical applications in order to achieve high noise immunity, low static power consumption, high yield, and good reliability [2]. Currently, SnO has been regarded as the most promising *p*-type oxide semiconductor due to its high stability in air and field-effect mobility in comparison to copper oxide Cu_2_O [4]. SnO exhibits excellent *p*-type conductivity due to the effective overlap of Sn 5*s* orbitals at the valance band maximum [8]. Different deposition techniques, including thermal evaporation [9,10], electron beam evaporation [11], pulsed laser deposition [12,13], and RF/DC sputtering [14,15,16], have been used to obtain *p*-type SnO. Among these techniques, sputtering is more desirable as it is widely used in industries for thin-film deposition [17]. A field-effect hole mobility above 10 cm^2^/(V∙s) has been realized for SnO TFTs [15], which is comparable to the typical values obtained by *n*-type oxide semiconductors such as IGZO and ZnO. This makes SnO a suitable candidate for future thin-film complementary electronics. So far, CMOS inverters using *n*-type semiconductors (such as SnO_2_, ZnO) and *p*-type SnO TFTs have been fabricated [18,19,20,21,22]. CMOS-like inverters based on bipolar SnO TFTs have also been demonstrated [12]. From an applications point of view, it may be highly desirable to complement SnO with IGZO for CMOS logic gates because both semiconductors can be deposited by sputtering techniques, the same method widely used in the current IGZO electronics industries. Their mechanical flexibility and high transparency in the visual region are also preferable for future flexible and transparent electronic devices, which are regarded as the basis of the Internet of Things. In this work, we fabricated *n*-type IGZO and *p*-type SnO TFTs. The *p*-type SnO TFTs were optimized through the use of different thermal treatments of the SnO thin-films. Complementary inverters based on IGZO and SnO TFTs were demonstrated with a full output voltage swing. By cascading the inverters with large noise margins, a three-stage IGZO and SnO ring oscillator was fabricated to operate at 2.63 kHz with a high output amplitude. The incorporation of IGZO and SnO into CMOS logic offers a promising route towards flexible CMOS electronics, such as radio-frequency identification tags and fully oxide-based microprocessors.

## 2. Materials and Methods

The TFTs were fabricated on highly *p*-type doped Si substrate with 100-nm-thick thermally oxidized SiO_2_. By using a 3 in metallic Sn target, a 27-nm-thick SnO film was deposited by using RF sputtering at 150 W in the Ar/O_2_ mixture gas. The pressure was 4.6 mTorr and the flow rates of Ar and O_2_ were 21 sccm and 3 sccm, respectively. Then the SnO films were thermally annealed in air at different temperatures from 100 °C to 250 °C for 1 h. The 50-nm-thick Pt source/drain contacts were deposited by RF sputtering at 80 W in Ar. 

For the *n*-type TFTs a 24-nm-thick IGZO active layer was deposited by using RF sputtering at 80 W with a pressure of 4.2 mTorr. The atomic ratio for the target was In:Ga:Zn = 1:1:1. Titanium source/drain contacts were deposited by E-beam evaporator for 50 nm.

**Figure 1:**
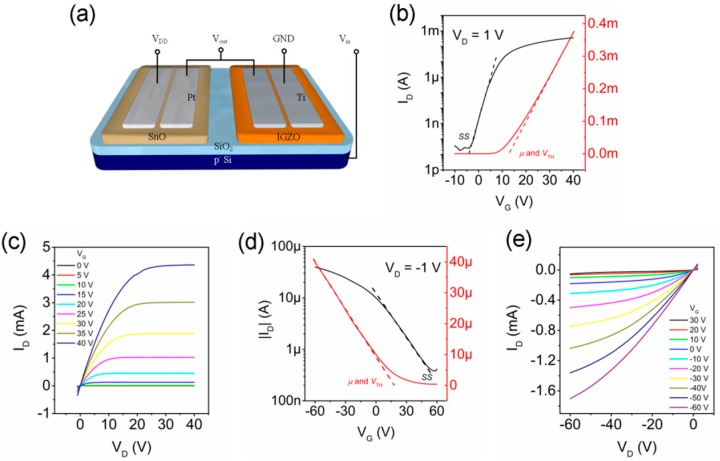
(**a**) Schematic of the SnO and IGZO TFTs; (**b**) Transfer and (**c**) output characteristics of the IGZO TFT; (**d**) Transfer and (**e**) output characteristics of the SnO TFT.

The channel width and the channel length of the TFTs were 2 mm and 60 µm, respectively. All patterns were defined by shadow masks as shown in Figure 1a. The electrical characteristics were measured by using Agilent E5260B at room temperature in dark. The output of the ring oscillator was measured by using Agilent 54622A oscilloscope.

## 3. Results and Discussion

Figure 1b,c show the transfer and output characteristics of the IGZO TFTs. The device demonstrated an on/off ratio higher than 10^7^. In the output curves, the good linear regions at low drain voltages (*V_D_*) indicate Ohmic contact between Ti and IGZO. In order to obtain high-quality *p*-type SnO films, post-annealing treatments at temperatures below 300 °C are normally required [12,13,14,15]. In this work, different annealing temperatures were tested on SnO TFTs as shown in Supplementary Figure S1. The as-deposited film showed high conductivity and no field-effect modulation. After annealing at 150 °C, the device started to exhibit a weak *p*-type gate dependence, indicating the formation of a *p*-type channel. The device performance further improved after annealing at a higher temperature. It is found that the device annealed at 225 °C showed the best overall performance, and also demonstrated good air stability (Supplementary Figure S2). However, for the device annealed at 250 °C, the off-current increased almost one order of magnitude, which may be due to the disproportionation reaction of SnO (4SnO → Sn_3_O_4_ + Sn → 2SnO_2_ + 2Sn) [12]. In Figure 1d,e, the transfer and output characteristics of the SnO TFT annealed at 225 °C are presented. As the affinity and bandgap of SnO are estimated to be 3.59 eV [23] and 2.7–3.4 eV [24], in order to form good Ohmic contact with SnO, metals with a high work function need to be used as the source/drain contact. In this work, Pt with a work function of 5.4 eV [25] provides a good Ohmic contact according to the output curves shown in Figure 1e. In the linear transfer curve, the field-effect mobility, *µ*, and the threshold voltage, V_TH_, can be obtained by using

(1) $$I_{D}=\frac{W}{L}C_{ox}\mu \left(V_{G}-V_{TH}\right)V_{D},\;$$

where *I_D_* is the drain current; *W* and *L* are the channel width and length; *C_ox_* is the capacitance per unit area of the dielectric; *V_G_* is the gate voltage. As shown in Table 1, for the IGZO TFT, it was found that in the linear regime, the threshold voltage was 12.2 V and the linear mobility was 11.92 cm^2^/(V∙s). For the SnO TFT, the linear mobility was found to be 0.51 cm^2^/(V∙s). The threshold voltage was 26.3 V which is higher than that of the IGZO TFT, making it possible to form a high-performance complementary inverter by using SnO and IGZO TFTs. Another important parameter to describe the performance of TFTs is called subthreshold swing (*SS*), given by

(2) $$SS=ln10\frac{dV_{G}}{d\left(lnI_{D}\right)}=ln10\frac{k_{B}T}{q}\frac{N_{t}}{C_{ox}},$$

where *k_B_* is the Boltzmann constant; *T* is the temperature; *q* is the electron charge; and *N_t_* is the total interface trap density (an indicator of the interface and bulk trap densities) in the channel layer. In the IGZO TFT, the subthreshold swing (*SS*) was 1.84 V/dec which is slightly high due to the thick SiO_2_ dielectric layer. The extracted total interface trap density was 6.41 × 10^12^ cm^−2^·eV^−1^, which is comparable with the values found in other studies [26]. The extracted total interface trap density of the SnO TFT was found to 1.03 × 10^13^ cm^−2^·eV^−1^, which is typical for SnO TFTs [12,27] but still much higher than the value obtained in the IGZO TFT. This might be due to the polycrystalline structure and the multi-phases in the SnO active layer [2,15]. Such a high trap density also makes the Fermi level difficult to move up at positive biases, resulting in a low on/off ratio of 102 [13].

**Figure 2:**
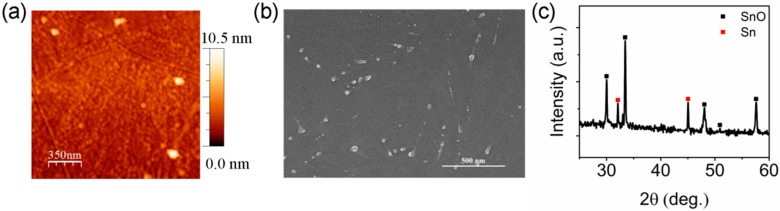
(**a**) AFM surface morphology, (**b**) SEM image, and (**c**) XRD pattern of the SnO film after being annealed in air at 225 °C for 1 h.

Figure 2a shows the surface morphology of an annealed SnO film which was fabricated under the same conditions as the SnO in the TFT. The root-mean-square roughness was 0.83 nm. The grain size was estimated to be around 50 nm, which might contribute to a better hole conduction as the grain boundaries would restrain the electron transport [9]. In both the AFM image and the scanning electron microscopy (SEM) image shown in Figure 2b, there are some clusters formed on the SnO film, which appear to be metallic Sn grains [27]. These clusters are formed after annealing as they cannot be seen in the SEM image of the as-deposited film (shown in the Supplementary Figure S3). Since the as-deposited SnO film was highly conductive, it is possible that such high conductivity is caused by the excess metallic Sn continuously distributed throughout the film [27]. After annealing, the formation of discontinuous Sn clusters may help to reduce the off-current. The XRD pattern of the 1-µm-thick SnO film in Figure 2b confirms the existence of metallic Sn. In the SnO films annealed at 225 °C, the excess Sn can exist in two forms, Sn clusters and interstitial tin atoms, in the SnO lattice [8,27]. The discontinuous Sn clusters should not affect the electrical performance of the SnO TFT. However, according to first principles calculations, interstitial tin atoms may result in a higher mid-gap trap density and thus lower the on/off ratio [8]. As these traps are sensitive to the stoichiometry of the SnO film, the device performance can be further improved by using different sputtering conditions and post-treatments [27,28].

**Figure 3:**
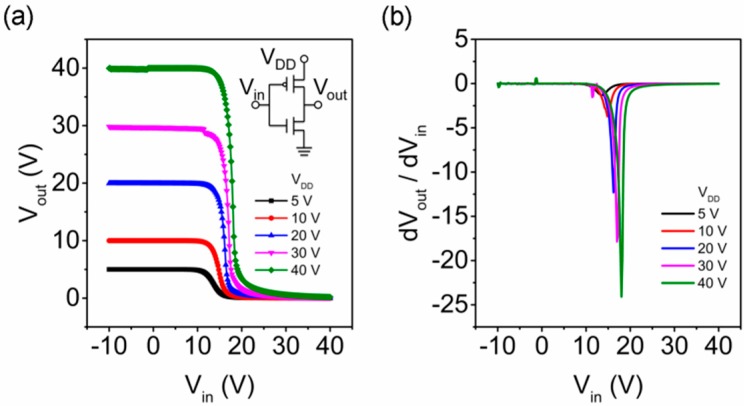
(**a**) Output voltage and (**b**) gain of the complementary inverter as a function of input voltage at different *V_DD_*.

The operation of the IGZO and SnO complementary inverter at different supply voltages, *V_DD_*, is shown in Figure 3a. The device demonstrated a full output voltage swing from 0 V to *V_DD_*. The threshold voltage was found to be 17 V where *V_in_* = *V_out_*. It is close to the ideal value, *V_DD_*/2. The input-low voltage (*V_IL_*) and the input-high voltage (*V_IH_*) are defined as the point where d(*V_out_*)/d(*V_in_*) = −1. It was found that *V_IL_* = 14.4 V and *V_IH_* = 20.0 V. The transition region can be determined by (*V_IH_* − *V_IL_*) which equals 5.6 V. The noise margin high was found to be 20 V by using (*V_DD_* − *V_IH_*), which is 50% of *V_DD_*, and the noise margin low was 14.4 V which equaled *V_IL_*, around 36% of *V_DD_*. The large values suggest that the complementary inverter could withstand a high noise level. In Figure 3b, the gain of the inverter at *V_DD_* = 40 V was found to reach as high as 24 even with the same *W*/*L* ratio. Compared with the gain obtained by other complementary oxide-based inverters, for example, 10 for SnO and In_2_O_3_ [29], four for SnO and SnO/Cu_2_O [22], 17 for SnO and ZnO [21], the combination of SnO and IGZO TFTs shows a promising potential pathway towards the realization of fully oxide-based electronics. In Supplementary Figure S4, the leakage current, *I_DD_*, at *V_DD_* = 5 V was found to be 1.4 nA in the on-state and 2.5 μA in the off-state, corresponding to 9 nW and 12.5 μW static power consumption at the on- and off-states, respectively. The high leakage current in the off-state is due to the high off-current of the SnO TFT. This can be further improved by optimizing the device geometry such as the channel width and channel length. By using high-*κ* dielectrics or decreasing the thickness of the SiO_2_ layer, the operation voltage of the inverter can be further reduced to meet the supply voltage in standard integrated circuits.

**Figure 4:**
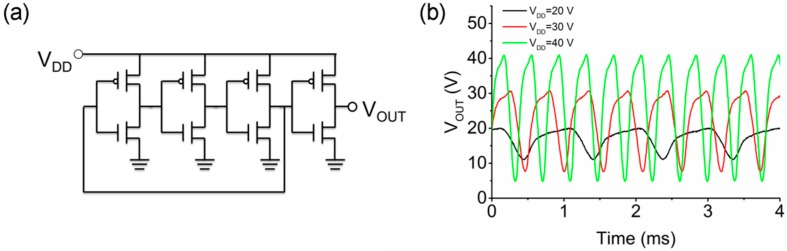
(**a**) Schematic of the three-stage ring oscillator with an output buffer; (**b**) Output voltage of the complementary ring oscillator as a function of time at different *V_DD_*.

As the maximum gain of the inverter was obtained at a positive input voltage, a ring oscillator can be built by directly stacking three inverters together as shown in Figure 4a. The extra inverter was used as a buffer to allow measurement of the output without disturbing the oscillation. The output voltage as a function of time is shown in Figure 4b at different supply voltages. The output amplitudes and frequencies are summarized in Table 2. It was found that the peak-to-peak amplitude and the frequency of the output signal at 40 V were 36.1 V and 2.63 kHz, respectively. Thus, the delay time for each stage was estimated to be 63 µs by using $f=1/\left(2nt_{d}\right)$, where n is the number of stages and $t_{d}$ represents the delay time. This value is similar to the propagation delay per stage of 50 μs in the SnO/ZnO ring oscillator [21]. However, the oscillation frequency of the complementary ring oscillator depends on the transit frequency of the TFT [30], which is proportional to $\mu /\left[L_{ch}\left(L_{ch}+L_{ol}\right)\right]$ where $L_{ch}$ is the channel length and $L_{ol}$ represents the length of the total overlapping area between the source/drain and the gate. In this work, $L_{ol}$ was 1.3 mm, significantly larger than the 25 μm used in the SnO/ZnO ring oscillator [21]. If scaling the device in this work down to the same dimensions as the bottom-gated IGZO pseudo-ring oscillator with a channel length of 10 μm, a 2 μm overlap and a $t_{d}$ of 136 ns [31], the propagation delay is estimated to be 92 ns. By further reducing $L_{ch}$ to 2 µm and $L_{ol}$ to 5 µm, it is possible to improve the oscillation frequency to 13.56 MHz, which could be used as the clock generator in oxide-semiconductor–based flexible radio-frequency identification tags. Despite that being the subject of future work, the demonstration of the complementary SnO/IGZO ring oscillator shows the potential of the fully oxide-based complementary electronics.

## 4. Conclusions

In this work, complementary inverters composed of the *n*-IGZO TFT and the *p*-SnO TFT were fabricated on Si substrates. The SnO TFT was annealed in air at 225 °C for 1 h to obtain *p*-type performance. The inverter demonstrated a gain of 24 at a supply voltage of 40 V. By cascading the inverters, a three-stage ring oscillator was demonstrated. The output frequency was 2.63 kHz with a supply voltage of 40 V. Our study suggests that other CMOS logic gates and more complex CMOS circuits can be made using SnO and IGZO TFTs in potential larger-area transparent electronics fully based on oxide semiconductors.

## Figures and Tables

**Table 1:** Electrical characteristics of the IGZO and SnO TFTs.

	IGZO TFT at *V_D_* = 1 V	SnO TFT at *V_D_* = −1 V
*µ* (cm^2^·V^−1^ s^−1^)	11.92	0.51
*V_TH_* (V)	12.21	20.11
*SS* (V/dec)	1.84	28.70
*N_t_* (cm^−2^ eV^−1^)	6.41 × 10^12^	1.03 × 10^13^
On/off ratio	10^7^	102

**Table 2:** Properties of the three-stage complementary ring oscillator.

*V_DD_*	Peak-Peak Amplitude	Measured Frequency
20 V	8.9 V	0.97 kHz
30 V	23.0 V	1.83 kHz
40 V	36.1 V	2.63 kHz

## Supplementary Materials

The following are available online at www.mdpi.com/1996-1944/10/3/319/s1, Figure S1. Transfer characteristics of the SnO TFTs annealed at different temperatures at *V_D_* = −1 V. Figure S2. Transfer characteristics of the SnO TFT annealed at 225 °C measured immediately after the fabrication and after six months in air, respectively. Figure S3. SEM image of the as-deposited SnO film. Figure S4. Output voltage and the leakage current of the inverter with *V_DD_* = 5 V.
