# Supplementary material for: High Performance Complementary Circuits Based on p-SnO and n-IGZO Thin-Film Transistors
Source: Materials (Basel). 2017 Mar 21;10(3):319. doi: 10.3390/ma10030319 (PMC5503323; doi:10.3390/ma10030319)
Supplement: Supplementary file 1 [file materials-10-00319-s001.pdf]

materials

Supplementary Materials: High Performance Complementary Circuits Based on p-SnO and n-IGZO Thin-Film Transistors

Jiawei Zhang, Jia Yang, Yunpeng Li, Joshua Wilson, Xiaochen Ma, Qian Xin and Aimin Song

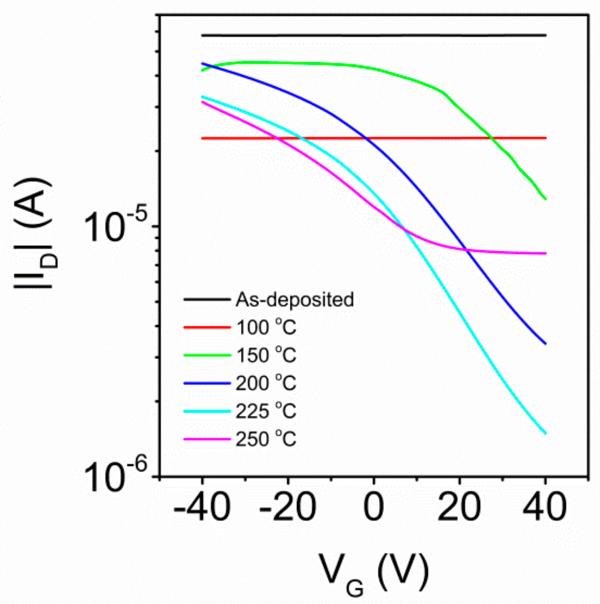

Figure S1. Transfer characteristics of the SnO TFTs annealed at different temperatures at V_D = -1 V.

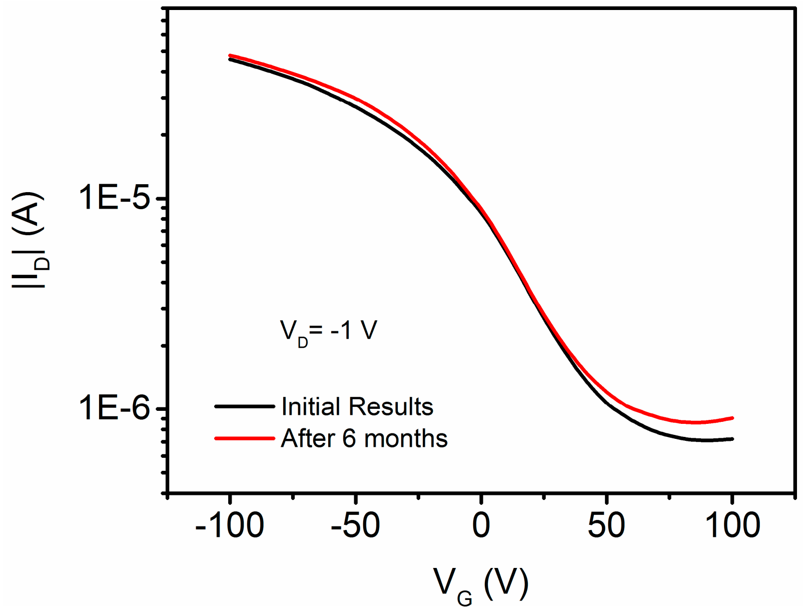

Figure S2. Transfer characteristics of the SnO TFT annealed at 225 °C measured immediately after the fabrication and after 6 months in air, respectively.

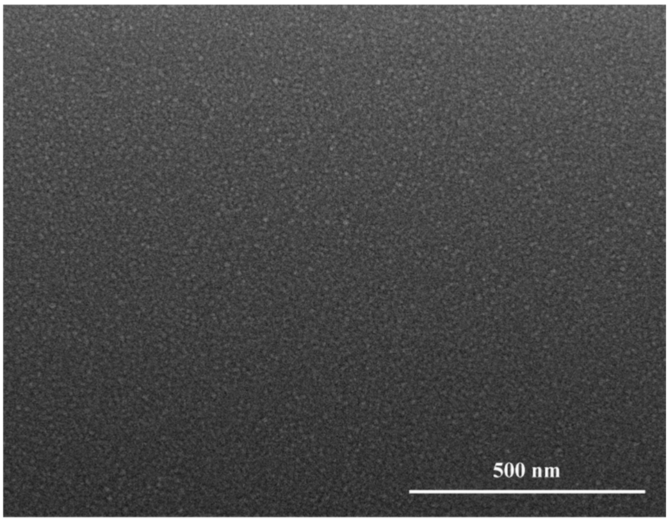

Figure S3. SEM image of the as-deposited SnO film.

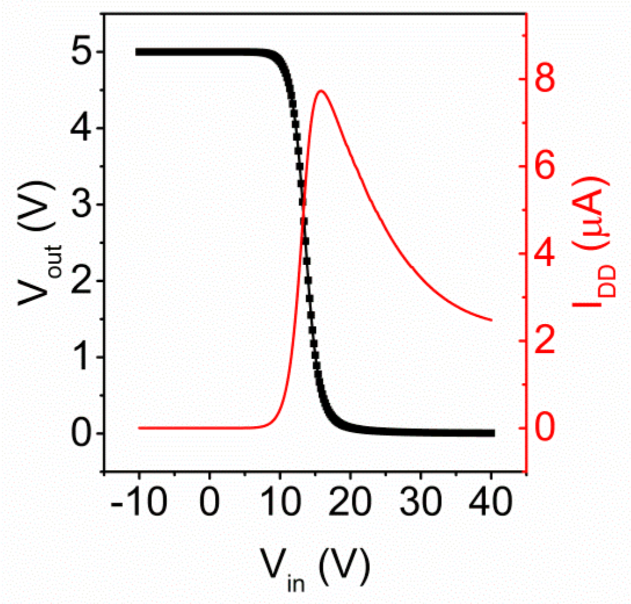

Figure S4. Output voltage and the leakage current of the inverter with $V_{DD} = 5$ V.
